# Risk factors for acquiring *Acinetobacter baumannii* infection in the intensive care unit: experience from a Moroccan hospital

**DOI:** 10.1099/acmi.0.000637.v3

**Published:** 2023-09-07

**Authors:** Elmostafa Benaissa, Elmehdi Belouad, Adil Maleb, Mostafa Elouennass

**Affiliations:** ^1^​ Department of Clinical Bacteriology, Mohammed V Military Teaching Hospital, Faculty of Medicine and Pharmacy of Rabat, Mohammed V University, Rabat, Morocco; ^2^​ Research Team of Epidemiology and Bacterial Resistance, Faculty of Medicine and Pharmacy of Rabat, Mohammed V University, Rabat, Morocco; ^3^​ Laboratory of Microbiology, Mohammed VI University Hospital, Faculty of Medicine and Pharmacy (University Mohammed the First), Oujda, Morocco

**Keywords:** *Acinetobacter baumannii*, infection, intensive care units, Risk factors

## Abstract

**Introduction.:**

*
Acinetobacter* species are non-fermenting and ubiquitous Gram-negative coccobacilli, which in recent years have become the leading cause of healthcare-associated infections worldwide. Our objective here was to study the epidemiology and risk factors associated with *Acinetobacter baumannii* infections in the intensive care unit (ICU).

**Methods.:**

This retrospective case-control study was conducted collaboratively between the Medical Bacteriology Department and the two ICUs of the Military Hospital of Instruction Mohammed V-Rabat over a 3 month period.

**Results.:**

We included 180 patients, of whom 60 had *A. baumannii* infection. We observed a male predominance in both matched groups, with a sex ratio of 1.6. The median age was 67 years [interquartile range (IQR) 59.5–77]. The median length of stay in the ICU before infection was 8.5 days (IQR 5–14). Multivariate logistic regression analysis identified the risk factors statistically associated with *A. baumannii* infection at the ICU level as follows: duration of invasive procedures >7 days [odds ratio (OR)=1.02], parenteral nutrition (OR=3.514), mechanical ventilation (OR=3.024), imipenem (OR=18.72), colistin (OR=5.645), probabilistic antibiotic therapy >4 days (OR=9.063) and neoplastic pathology (OR=5.727).

**Conclusion.:**

Based on our results, it can be inferred that shortening the duration of stay in the resuscitation setting, implementing rational use of medical devices and optimizing antibiotic therapy could decrease the incidence of these infections.

## Data Summary

No data were reused or generated for this study.

## Introduction

*Acinetobacter* is a ubiquitous, non-fermenting Gram-negative coccobacillus that has become the leading cause of healthcare-associated infections worldwide in recent years due to its ability to survive in the hospital environment and to acquire resistance to commonly used antibiotics. It has been implicated in a wide range of conditions including skin and soft tissue infections, pneumonia, osteomyelitis and bacteraemia.

The emergence of multi-drug-resistant *Acinetobacter baumannii* complicates the therapeutic process and contributes directly to the increase in mortality and indirectly to the increase in length of stay and hospitalization costs. In Moroccan intensive care units (ICUs), *Acinetobacter* species accounted for 24.85 % of all isolates and 31.5 % of all Gram-negative bacilli [1]. A recently published study demonstrated that the clonal spread of *A. baumannii* clinical isolates was related to those isolated from the hospital environment in two Moroccan ICUs [2]. The same research team also reported that clinical isolates of *A. baumannii* were more resistant to antiseptics and disinfectants than those from the environment [3].

This species has also become a matter of great concern due to its extraordinary ability to acquire resistance to commonly used antibiotics. Polymyxins remain the last therapeutic option but the emergence of colistin-resistant *A. baumannii* isolates has been reported worldwide [4]. Over the last decade, antibiotic resistance rates of *A. baumannii* strains have increased from 78.3 to 100 % for piperacillin/tazobactam, from 68.7 to 100 % for ceftazidime, from 31.4 to 87.7 % for imipenem, from 27.3 to 100 % for amikacin, and from 77.8 to 100 % for ciprofloxacin in Moroccan ICUs [1, 5, 6].

According to the literature, the recognized risk factors associated with *A. baumannii* infections are: invasive procedures, previous hospitalization, host factors, length of stay in the ICU and previous use of broad-spectrum antibiotics [4]. These infections are associated with mortality ranging from 28.3 to 84.3 % in the ICU [5, 7].

Independent predictors of mortality vary from country to country and region to region, and may be related to ICU-acquired infections, ineffective empirical antibiotic therapy, antibiotic resistance, immunosuppression, septic shock, medical device use and steroid use [7–10]. Local data are monocentric and fragmentary and sometimes without separation between services. It is in this context that we conducted this study with the objective of studying the epidemiology and risk factors associated with *A. baumannii* infections in the ICU of the Mohammed V Military Training Hospital.

## Methods

### Study design and study site

This is a retrospective case-control study conducted in collaboration between the medical bacteriology department and the two resuscitation departments of the Mohammed V military training hospital in Rabat over a 3 month period from June to September 2022.

### Study population

The original population consisted of patients hospitalized in the two series of medical and surgical resuscitation departments of the Military Hospital Mohammed V Rabat and having a positive biological workup for diagnostic purposes.

For our case-control study, 60 cases and 120 controls were randomly matched in a 1 : 2 ratio. Cases and controls were selected by including patients aged over 15 years, hospitalized in the two ICUs of the same facility and during the same study period. We excluded patients aged under 15 years of age, duplicates and patients whose records were lost.

Cases were defined as any patient infected with *A. baumannii* according to the Centers for Disease Control and Prevention criteria. Infection was considered acquired in the ICU if it occurred 48 h after admission to the ICU. Controls were defined as patients hospitalized in the ICU without *A. baumannii* infections. Clinical and microbiological data were collected from patient records and the laboratory information system using a questionnaire with the following variables: age, gender, patient’s origin, underlying comorbidities, invasive procedures, previous probabilistic antibiotic therapy, notion of prior hospitalization up to 1 year before inclusion, length of stay in an ICU setting, bacterial co-infection, microbiological data (antibiogram, sampling site, type of sampling) and evolution.

### Microbiological method

Identification of bacterial isolates was based on culture, morphological and biochemical identification characteristics. Biochemical identification was performed using API20NE ready-to-use galleries (bioMérieux).

Antibiotic susceptibility was studied using the Mueller Hilton agar diffusion method by using OXOID-type antibiotic discs and interpreted according to the recommendations of EUCAST 2022. The interpretation was performed using the Adagio Biorad system. Quality control of the antibiotic susceptibility test was performed with *Escherichia coli* strain ATCC 25922.

For colistin, determination of the MIC was performed by the microdilution method using the sensititre COL* plate (Thermo Scientific Wellwash; RRID:SCR_020569) from an 18–24 h culture. Interpretation of the results was performed according to EUCAST 2022 recommendations.

### Statistical methods

Data were entered into Microsoft Excel, RRID:SCR_016137. Statistical analysis was performed using Jamovi version 2.3.4. Quantitative variables were expressed as mean±sd or median (interquartile range – IQR) and qualitative variables as number and percentage. Comparison of qualitative variables was performed by Pearson’s and Fisher’s chi-square exact tests, and comparison of quantitative variables was performed by Student’s t-test and Mann–Whitney U-test according to the normality of the distribution. Multivariate analysis was performed using a logistic regression model.

Measures of association were calculated with 95 % confidence intervals by setting a threshold of statistical significance at *P*≤0.05.

## Results

### Patient characteristics

We included 180 patients meeting the eligibility criteria, 60 of whom had *A. baumannii* infection. We noted a male predominance in both matched groups with a sex ratio of 1.6. The median age of our population was 67 years (IQR 59.5–77). The median length of stay in the ICU before infection was 8.5 days (IQR 5–14).

Among 420 patients hospitalized in the ICU during the study period, 60 developed *A. baumannii* infection. The incidence of *A. baumannii* care-associated infections was 14.2 per 100 patients.

Pulmonary infections were frequent in 60 % (46/60), followed by bacteraemia in 23 % (14/60), urinary tract infections in 20 % (12/60) and surgical site infections in 8.3 % (5/60). The reason for hospitalization in our series was dominated by respiratory distress in 30 % (55/180), followed by septic shock in 10 % (18/180), stroke in 9.4 % (17/180), postoperative management in 9.4 % (17/180), disturbed consciousness in 7.2 % (13/180) and haemodynamic instability in 5.5 % (10/180).

Co-infection was found in 75 % (45/60). The most responsive co-isolates were *Klebsiella pneumoniae* in 18.3 % (11/60), followed by *Escherichia coli* in 11.7 % (7/60), *Enterococcus* sp. in 10 % (6/60), *Corynebacterium* spp. in 8.3 % (5/60) and *Enterobcater cloacae* in 8.3 % (5/60) (Table 1).

**Table 1.** The distribution of bacterial co-infection in patients with *A. baumannii* infection

Species	No.	Percentage
* Klebsiella pneumoniae *	11	18.3 %
* Escherichia coli *	7	11.7 %
* Enterococcus* sp*.*	6	10 %
* Enterobacter cloacae *	5	8.3 %
* Corynebacterium* spp*.*	5	8.3 %
* Pseudomonas aeruginosa *	4	6.7 %
* Serratia* sp*.*	2	3.3 %
Coagulase-negative *Staphylococcus *	2	3.3 %
* Haemophilus influenzae *	1	1.7 %
*Streptococcus agalactia*	1	1.7 %
* Staphylococcus aureus *	1	1.7 %

**Fig. 1.**
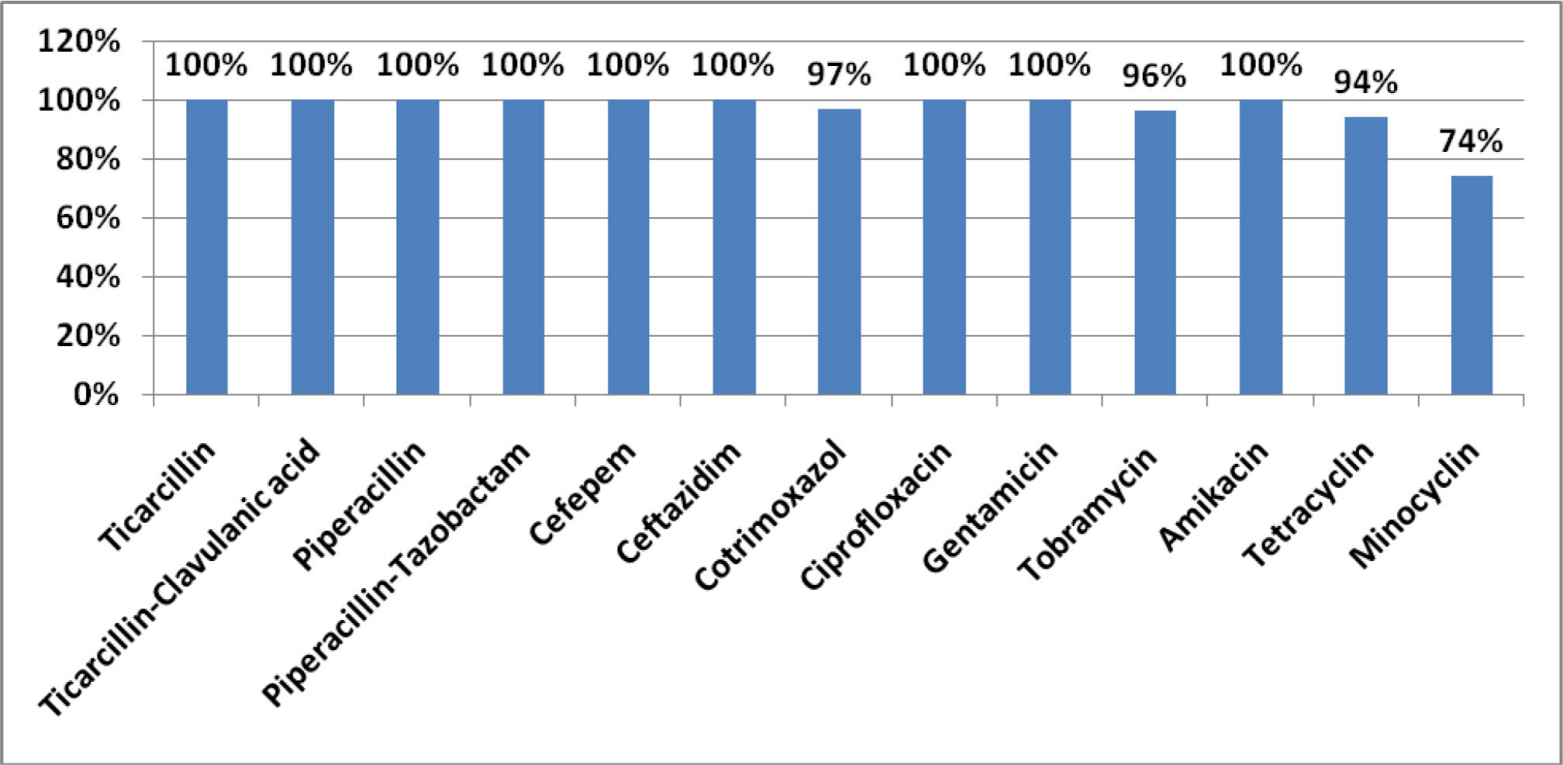
Antibiotic resistance profile of *Acinetobacter baumannii* isolates.

Study of the resistance of *A. baumannii* isolates to antibiotics showed resistance rates of 100 % for ticarcillin, piperacillin, piperacillin-tazobactam, ticarcillin-clavulanic acid, cefepime, ceftazidime, gentamicin, amikacin, imipeneme and ciprofloxacin. Tetracylin and minocyclin were resistant in 94 and 74% of isolates respectively. All isolates of *A. baumannii* were susceptible to colistin. Following the results of the resistance study, we deduced that all our isolates were represented by extremely drug-resistant *A. baumannii* (XDR) (Fig. 1).

### Risk factors for *A. baumannii* infection in the resuscitation setting

Variables found to be statistically significant in univariate analysis were: disorders of consciousness, duration of invasive procedures >7 days, previous use of central venous catheters, arterial catheters, mechanical ventilation, parenteral nutrition, co-infection, probabilistic antibiotic therapy, probabilistic antibiotic therapy >4 days, previous use of colistin, imipenem, documented antibiotic therapy, clinical course, length of stay in the ICU and length of stay in the ICU >14 days (Table 2).

**Table 2.** Results of univariate analysis of potential risk factors for *Acinetobacter baumannii* infection in the resuscitation setting

Characteristic	Cases (*n*=60)	Controls (*n*=120)	*P*	OR	95 % CI
Male sex	37(62 %)	78 (65 %)	0.661	0.866	0.785–1.498
Age in years, mean±sd and median (IQR)	67.2±13.4	69 (60.5–77)	0.520	0.994	0.260–1.013
Duration of invasive procedures in days, median (IQR)	13(8.75–18.3)	8 (5–10)	<0.001	1.134	0.067–1.185
Length of stay in the intensive care unit in days, median (IQR)	14 (9–19)	7 (5–10)	<0.001	1.132	0.068–0.181
Length of stay in intensive care in days >14 days	31 (51.7 %)	16 (13.3 %)	<0.001	6.948	1.21–8.669
Stay in another department	33 (55 %)	70 (58.3 %)	0.670	0.873	0.761–1.489
**Reason for hospitalization**					
Respiratory distress	12 (20 %)	21 (17.5 %)	0.647	0.853	0.839–1.522
Consciousness disorder	8 (13.3 %)	5 (4.17 %)	0.033	3.538	0.99–4.428
Haemodynamic instability	4 (6.7 %)	6 (5 %)	0.646	1.357	1.000–1.610
Aceto-ketotic decompensation	3 (5 %)	4 (3.3 %)	0.588	1.526	1.11–1.953
Cerebral vascular accidents	6 (10 %)	11 (9.2 %)	0.857	1.101	0.951–2.143
COVID-19	4 (6.7 %)	3 (2.5 %)	0.190	2.786	0.506–3.555
Meningitis syndrome	5 (1.7 %)	3 (2.5 %)	0.091	3.545	0.201–2.732
Post-operative management	3 (5 %)	14 (11.7 %)	0.161	0.398	2.208–0.368
Sepsis	3 (5 %)	13 (10.8 %)	0.599	0.748	1.371–0.791
Status epilepticus	2 (3.3 %)	1 (0.83 %)	0.732	1.234	0.421–2.156
**Invasive procedures**					
Duration of invasive procedures >7 days	48	75	0.019	2.400	0.143–1.608
Central venous catheter	58	66	0.05	4.462	0.009–6.000
Arterial catheter	45	55	˂0.001	3.545	0.580–4.952
Urinary catheter	57	112	0.661	1.357	0.06–1.670
Nasogastric catheter	47	49	0.351	1.021	0.1-1.423
Mechanical ventilation	49	72	0.004	2.970	0.339–3.837
Parenteral nutrition	27	19	˂0.001	4.349	0.764–6.176
Dialysis	2	2	0.483	2.034	1.27–3.695
Co-infection	44	60	0.003	2.750	0.337–3.686
Recent surgery	3	7	0.818	0.850	0.55–1.227
Probabilistic antibiotic therapy	59	106	0.05	7.792	1.20–9.107
Probabilistic antibiotic therapy >4 days	52	89	0.05	2.264	0.032–3.666
Previous use of penicillins+inhibitors	10	25	0.506	0.760	0.084–1.535
Use of C3Gs and C4Gs	26	53	0.915	0.967	0.659–1.591
Use of aminoglycosides	22	43	0.913	1.037	0.608–1.680
Use of flouroquinolones	9	13	0.423	1.452	0.540–2.286
Use of colistin	38	4	˂0.001	50.091	20.79–60.04
Use of glycopeptides	4	6	0.646	1.357	1.00–1.610
Use of imipenem	29	39	0.04	1.943	0.029–2.298
Documented antibiotic therapy	28	3	˂0.001	34.125	8.28–48.783
Deaths	18	19	0.029	1.439	0.562–2.085
Underlying pathologies	38	91	0.08	0.550	0.268–1.074
Diabetes	19	37	0.909	1.040	0.629–1.707
Hypertension	20	44	0.660	0.864	0.799–1.506
Cerebral vascular accidents	4	6	0.646	1.357	0.10–1.610
Chronic smoking	7	17	0.642	0.800	0.163–1.718
Heart disease	4	8	1.00	1.00	0.24–1.242
Chronic obstructive pulmonary disease	1	4	0.529	0.492	0.024–1.503
Cancer	11	12	0.119	2.020	0.182–2.588
Epilepsy	3	2	0.222	3.105	0.684–3.950

*CI, confidence interval; OR, odds ratio.

C3Gs and C4Gs, 3rd and 4th generation cephalosporins; CI, confidence interval; COVID-19, Coronavirus disease 2019; IQR, interquartile range; OR, odds ratio; SD, standard deviation.

Therefore, multivariate logistic regression analysis with the ‘backward’ top-down variable selection method identified risk factors statistically associated with *A. baumannii* infection at the ICU level: duration of invasive procedures >7 days [odds ratio (OR)=1.02], parenteral nutrition (OR=3.514), mechanical ventilation (OR=3.024), imipenem (OR=18.72), colistin (OR=5.645), probabilistic antibiotic therapy >4 days (OR=9.063) and neoplastic pathology (OR=5.727) (Table 3).

**Table 3.** Results of multivariate logistic regression analysis of risk factors associated with *Acinetobacter baumannii* infection

Parameter	*P*	OR	95 % CI
Duration of invasive procedures ˃7 days	0.011	1.42	0.825–1.900
Parenteral nutrition	0.019	3.514	0.591–6.528
Imipenem	0.006	18.725	1.495–8.970
Mechanical ventilation	0.031	3.024	0.873–6.320
Colistin	˂0.001	5.645	5.07–16.811
Probabilistic antibiotic therapy ˃4 days	0.001	9.063	3.538–14.686
Neoplastic pathology	0.006	5.727	1.816–10.884

CI, confidence interval; OR, odds ratio.

### Risk factors associated with mortality

Regarding the mortality rate, 30 % of patients infected with *A. baumannii* died during their hospitalization compared to 16 % in the control groups (*P*<0.029).

Univariate analysis suggested that the following were risk factors for mortality: *A. baumannii* infection, duration of probabilistic antibiotic therapy <4 days, chronic smoking and neoplastic pathology (Table 4).

**Table 4.** Results of univariate analysis of risk factors for mortality in patients infected with *Acinetobacter baumannii
*

Characteristic	Deaths (*n*=37)	Survivors (*n*=143)	*P*	OR	95 % CI
Male sex	28	87	0.098	0.499	0.52–1.128
Age in years, median (IQR)	67 (60–76)	68 (60–76)	0.927	0.999	0.024–1.022
Duration of invasive procedures in days, median (IQR)	9 (5–17)	9 (6–12)	0.850	0.996	0.047–1.003
Length of stay in the intensive care unit in days, median (IQR)	9 (5–18)	8 (5.5–13.5)	0.956	0.999	0.043–1.04
Length of stay in intensive care in days >14 days	11	36	0.574	1.26	0.571–2.03
Stay in another department	21	82	0.949	1.02	0.706–1.754
**Reason for hospitalization**					
Respiratory distress	13	42	0.498	1.30	0.501–2.03
Consciousness disorder	4	9	0.350	1.80	0.647–2.83
Aceto-ketotic decompensation	1	6	0.678	1.58	1.693–2.60
Cerebral vascular accidents	2	15	0.355	2.05	0.804–2.24
COVID-19	2	5	0.595	1.58	1.226–2.14
Meningitis syndrome	1	7	0.570	1.85	1.510–2.74
Post-operative management	3	14	0.756	1.23	1.096–1.51
Sepsis	6	12	0.165	2.11	0.307–3.80
**Invasive procedures**					
Duration of invasive procedures >7 days	25	98	0.911	1.05	0.729–1.818
Central venous catheter	31	131	0.165	0.473	0.80–1.307
Arterial catheter	21	79	0.869	1.06	0.668–1.791
Urinary catheter	33	136	0.192	2.35	0.429–3.14
Nasogastric catheter	20	76	0.921	0.964	0.762–1.689
Mechanical ventilation	22	99	0.261	1.53	0.318–2.12
Parenteral nutrition	7	39	0.302	1.61	0.427–2.38
Co-infection	24	80	0.329	1.45	0.377–2.13
Recent surgery	1	9	0.410	2.42	1.215–3.98
Probabilistic antibiotic therapy >4 days	24	117	0.029	2.4	0.093–3.69
Previous use of penicillins+inhibitors	11	24	0.08	2.10	0.089–2.57
Use of C3Gs and C4Gs	14	65	0.406	1.37	0.427–2.06
Use of aminoglycosides	15	50	0.530	1.27	0.503–1.978
Use of flouroquinolones	3	19	0.396	1.74	0.723–2.83
Use of colistin	9	33	0.873	1.07	0.777–2.915
Use of glycopeptides	1	9	0.410	2.42	0.215–2.98
Use of macrolides	2	4	0.439	0.504	0.42–1.05
Use of imipeneme	14	54	0.993	1	0.742–1.749
Documented antibiotic therapy	9	22	0.203	1.77	0.308–2.45
* Acinetobacter baumannii* infection	18	42	0.029	2.28	0.085–2.56
Underlying pathologies	26	103	0.883	1.09	0.708–2.881
Diabetes	12	44	0.846	1.08	0.698–1.851
Hypertension	10	54	0.227	1.64	0.307–2.29
Cerebral vascular accidents	2	8	0.964	1.04	0.557–2.63
Chronic smoking	9	15	0.032	2.74	0.087–3.93
Heart disease	1	10	0.731	1.32	0.289–2.89
Chronic obstructive pulmonary disease	2	2	0.293	2.67	0.846–3.81
Cancer	9	14	0.022	2.96	0.154–3.02

C3Gs and C4Gs, 3rd and 4th generation cephalosporins; CI, confidence interval; COVID-1, Coronavirus disease 2019; IQR, interquartile range; OR, odds ratio; SD, standard deviation.

However, multivariate logistic regression with the variable selection method considering those risk factors with a *P*˂0.05, those with a *P*<0.3 and also those recognized in the literature associated with mortality by *A. baumannii* infection showed that *A. baumannii* infection, sepsis and probabilistic antibiotic therapy <4 days were significant independent predictors (*P*<0.005) (Table 5).

**Table 5.** Results of multivariate logistic regression analysis of risk factors for mortality in patients infected with *Acinetobacter baumannii
*

Parameter	*P*	OR	95 % CI
* Acinetobacter baumannii* infection	0.015	2.43	0.444–4.133
Sepsis	0.030	2.17	0.181–3.477
Probabilistic antibiotic therapy ˂4 days	0.038	2.07	0.073–2.557

CI, confidence interval; OR, odds ratio.

## Discussion

*A. baumanni* is one of the most common opportunistic agents causing healthcare-associated infections, especially in the ICU setting. In the absence of incontrovertible data on *A. baumannii* infections, it is difficult to accurately estimate the incidence and thus the prevalence of multidrug-resistant bacterial infections including multidrug-resistant *A. baumannii.*

In our study, the incidence of *A. baumannii* infections in the ICU setting was 14.2 %. Our rate is higher than that reported by Jean *et al.* (8.4 %), and that reported in India (10%) and in Mexico in cancer patients (4.6 %). This could be explained by differences in the application of hygiene and infection control measures, in particular hand hygiene practices and decontamination of the hospital environment [11].

In our study, independent risk factors for *A. baumannii* infection can be classified into three categories: those related to increased length of stay in the ICU, those related to the use of invasive medical devices (use of central venous catheters or mechanical ventilation and procedures ≥7 days), and those related to previous drug treatment (probabilistic antibiotic therapy >4 days, imipenem, colistin).

According to the literature, risk factors vary between countries and regions. The most frequently reported risk factors were previous exposure to carbapenems and previous antibiotic therapy [12–17].

A meta-analysis of 18 studies identified risk factors for multidrug-resistant bacterial infections. Male gender, exposure to surgery, central venous catheter, mechanical ventilation, previous antibiotic therapy and prolonged hospitalization were considered as risk factors for multidrug-resistant infections [18]. Following our multivariate analysis, mechanical ventilation, use of probabilistic antibiotic therapy >4 days, parenteral nutrition, duration of invasive procedures >7 days, colistin, imipenem and neoplastic pathologies were independent risk factors. Our case differs from previous studies in case definitions, anatomical site of infection, antibiotic treatment protocols and antibiotic resistance profile.

Mechanical ventilation is the major factor in the emergence of nosocomial pneumonia caused by multidrug-resistant *A. baumannii *. In China, a retrospective incidence study showed that the use of mechanical ventilation led to a 2.5-fold higher risk of developing a nosomial infection with multidrug-resistant bacteria [19]. In Greece, a prospective study showed that mechanical ventilation was a risk factor independently associated with the occurrence of healthcare-associated infections with multidrug-resistant bacteria. In our study, multivariate analysis showed that the use of mechanical ventilation increased the risk of acquiring healthcare-associated infections with multi-drug-resistant *A. baumannii* by three times compared to control patients [20]. Intravascular devices are essential to modern medicine in the management of patients but their presence is not without infectious risk. This explains why *A. baumannii* isolates are frequent in respiratory infections and bacteraemia.

Our data also showed that imipenem and colistin increased the risk of *A. baumannii* infection by 18 and five times, respectively.

Prior exposure to antibiotics has been identified as a risk factor for multidrug-resistant bacterial infection in several studies [21, 22]. This highlights the importance of rational use of antibiotics. In our study, probabilistic antibiotic therapy longer than 4 days increased the risk of acquiring *A. baumannii* infection nine times compared to the control group.

Furthermore, our data showed that neoplastic pathology increased the risk of *A. baumannii* infection by 5.7 times in the cases compared with in the control group.

In the present study, the observed mortality rate (30%) is comparable to the literature, which ranges from 29 to 63 % [23–25]. According to our study the risk of death is almost two times higher in patients infected with *A. baumannii* than in controls.

The literature is conflicting regarding the impact of bacterial resistance on mortality. A very high mortality rate has been reported in patients infected with extended-spectrum beta-lactamase (ESBL) enterobacteria, multidrug-resistant *A. baumannii* and multidrug-resistant *Pseudomonas aeruginosa *, while some studies have reported similar mortality rates for cases and controls. Several studies over the past two decades have demonstrated that inappropriate initial antimicrobial therapy is an independent risk factor for mortality in patients with multidrug-resistant bacteria [26–36].

Septic shock was statistically associated with two-fold greater mortality. Previous studies have shown that septic shock remains the leading cause of death in patients with multi-drug-resistant bacterial infection in the ICU and is a risk factor for mortality with inappropriate initial antimicrobial therapy [37]. Our data also showed that probabilistic antibiotic therapy of less than 4 days increases the risk of mortality by 2.07. Being infected with multidrug-resistant *A. baumannii* has been shown to be associated with a 2.43-fold increased risk of mortality.

## Conclusion

Our data show that reducing the duration of ICU stay, rational use of medical devices and optimizing the initial empirical antibiotic therapy could significantly reduce the incidence of these infections. Patients with *A. baumannii* infection and septic shock have a poor prognosis. A local antibiogram database is needed to better monitor the evolution of bacterial resistance in our hospital.

## Supplementary Data

Supplementary material 1
